# Supplementary material for: Biotransformed Metabolites of the Hop Prenylflavanone Isoxanthohumol
Source: Molecules. 2019 Jan 22;24(3):394. doi: 10.3390/molecules24030394 (PMC6385124; doi:10.3390/molecules24030394)
Supplement: Supplementary file 1 [file molecules-24-00394-s001.pdf]

Isoxanthohumol (1): white amorphous powder, $[\alpha]_D$ +0.0° (*c* 0.2, MeOH); UV λ_{max} (MeOH) (log ε) 286 (4.34), 397 (3.56); IR (KBr) ν_{max}: 3393, 1597, 1519, 1450, 1415, 1349, 1274, 1148, 1092, 834 cm^{-1}; ^{1}H NMR (CD_3OD, 500 MHz) δ 7.30 (2H, d, *J* = 8.5 Hz, H-2′, 6′), 6.82 (2H, d, *J* = 8.5 Hz, H-3′, 5′), 6.12 (1H, s, 1H), 5.26 (1H, dd, *J* = 12.8, 2.8 Hz, H-2), 5.13 (1H, brt, *J* = 7.2 Hz, H-2″), 3.79 (3H, s, 5-OC*H*3), 3.20 (2H, m, H-1″), 2.96 (1H, dd, *J* = 16.8, 13.0 Hz, H-3a), 2.66 (1H, dd, *J* = 16.8, 3.0 Hz, H-3b), 1.61 (3H, s, H-5″), 1.55 (3H, s, H-4″); ^{13}C NMR (CD_3OD, 125 MHz) δ 193.2 (C-4), 164.5 (C-8a) 164.0 (C-7), 162.0 (C-5), 158.9 (C-4′), 131.9 (C-3″), 131.7 (C-1′), 129.0 (C-2′,6′), 124.0 (C-2″), 116.0 (C-3′,5′), 110.1 (C-8), 105.9 (C-4a), 93.6 (C-6), 80.1 (C-2), 56.1 (5-O*C*H3), 46.3 (C-3), 26.1 (C-5″), 22.8 (C-1″), 18.1 (C-4″); EIMS 70eV *m/z* 354 $[M]^+$, 339, 311, 299, 234, 219, 191, 179, 120 (calcd. for $C_{21}H_{22}O_5$).

Figure S1. ^{1}H NMR spectrum of **1**

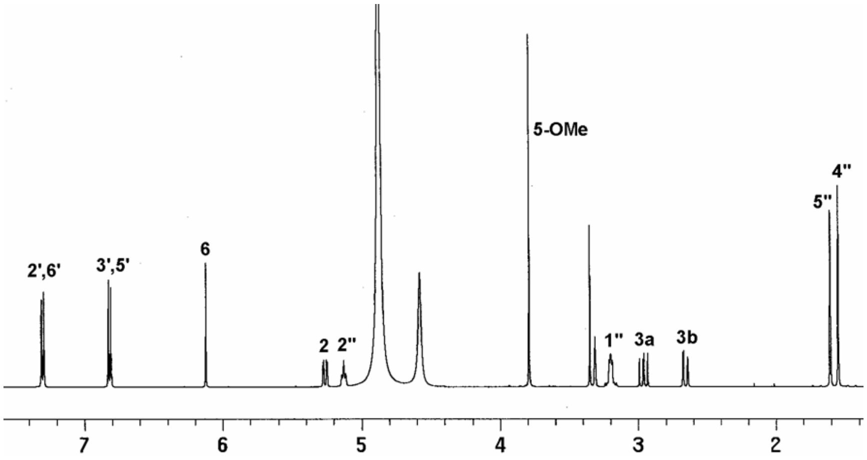

Figure S2. ^{13}C NMR spectrum of **1**

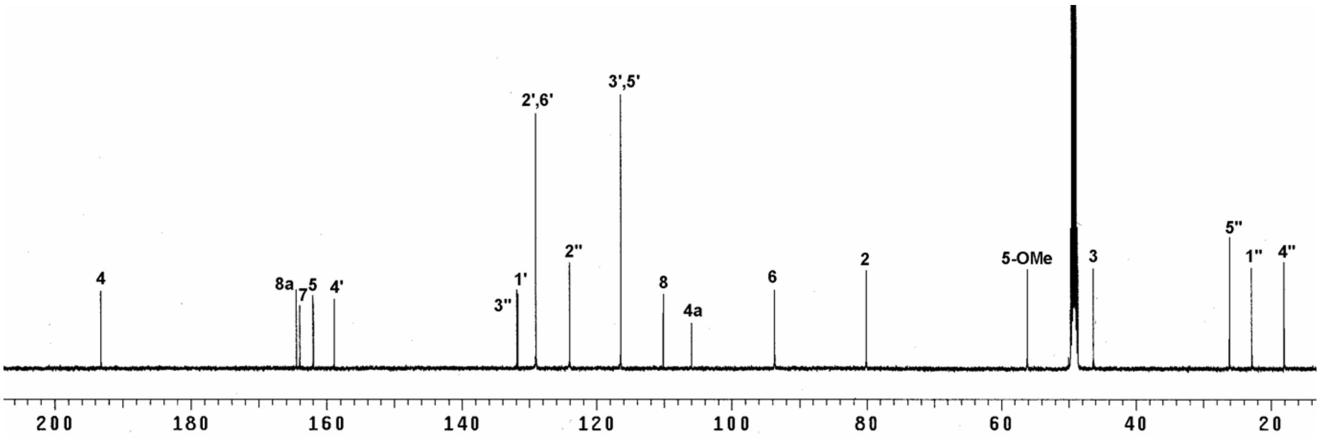

Scheme S1. HPLC profiles of oxygenated metabolites (**2-7**) of isoxanthohumol (**1**)

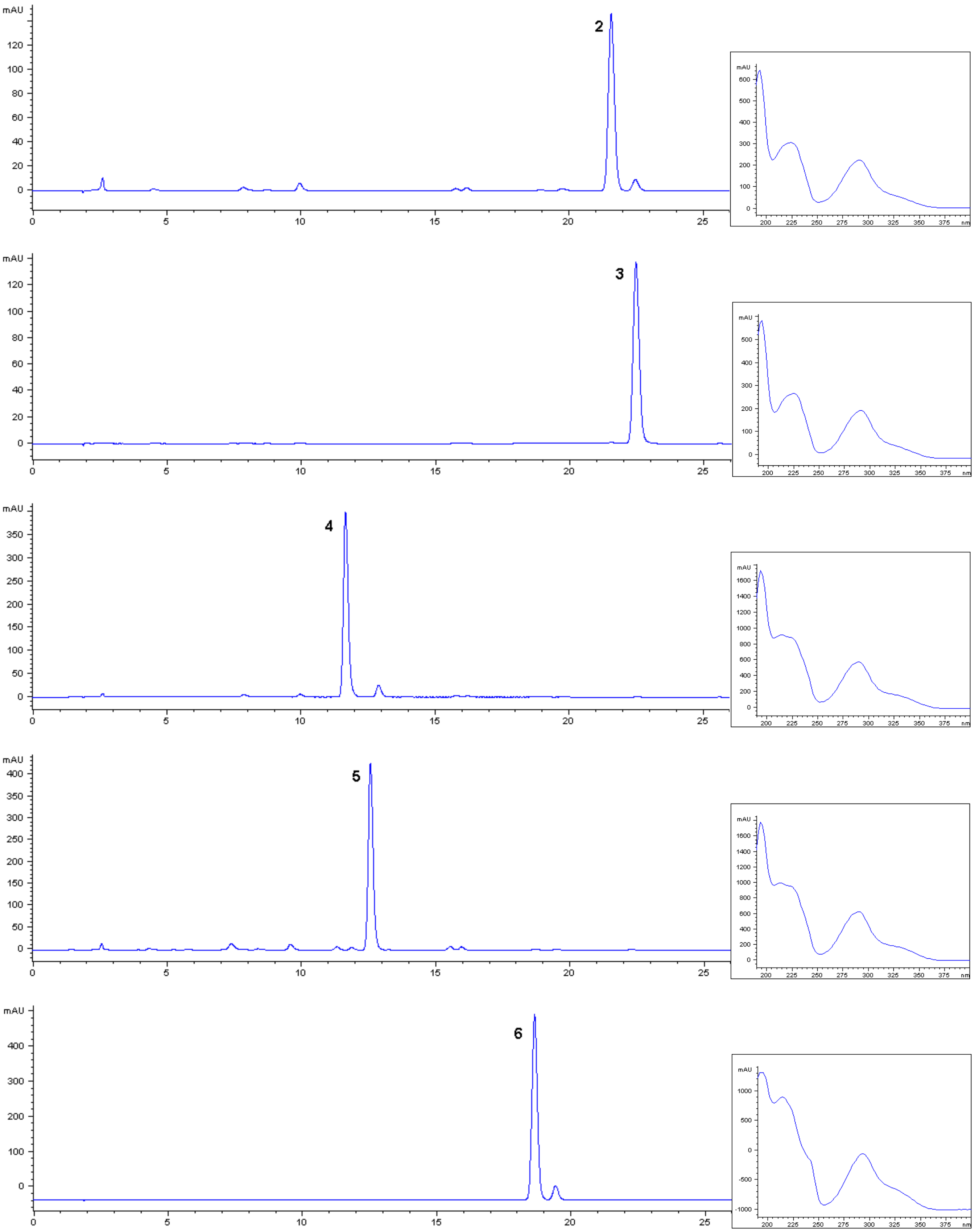

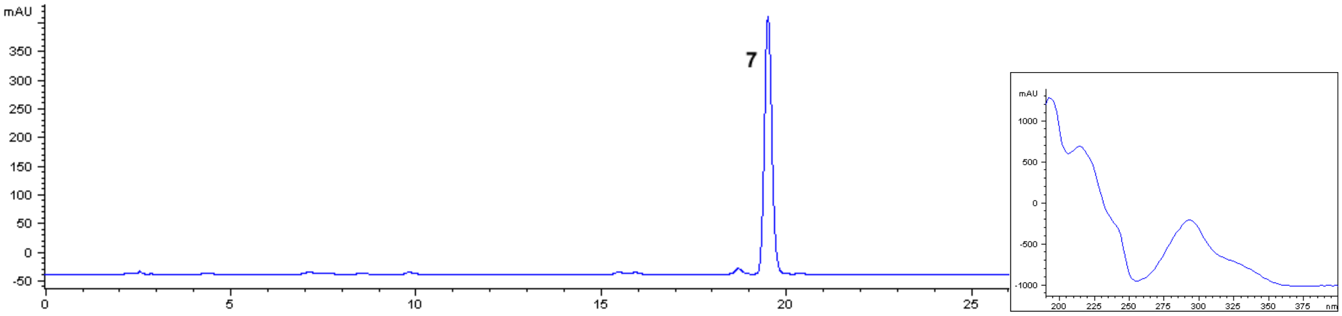

HPLC was performed on a Hewlett Packard 1100 series composed of a degasser, a binary mixing pump, a column oven and a DAD detector using Waters SunFire™ (4.6 × 150 mm, 5 μm) with acetonitrile (solvent A) and water containing 0.1% formic acid (solvent B) at 1 mL/min under the wavelength 280 nm.

Figure S3. ^{1}H NMR spectrum of the new compound **2**

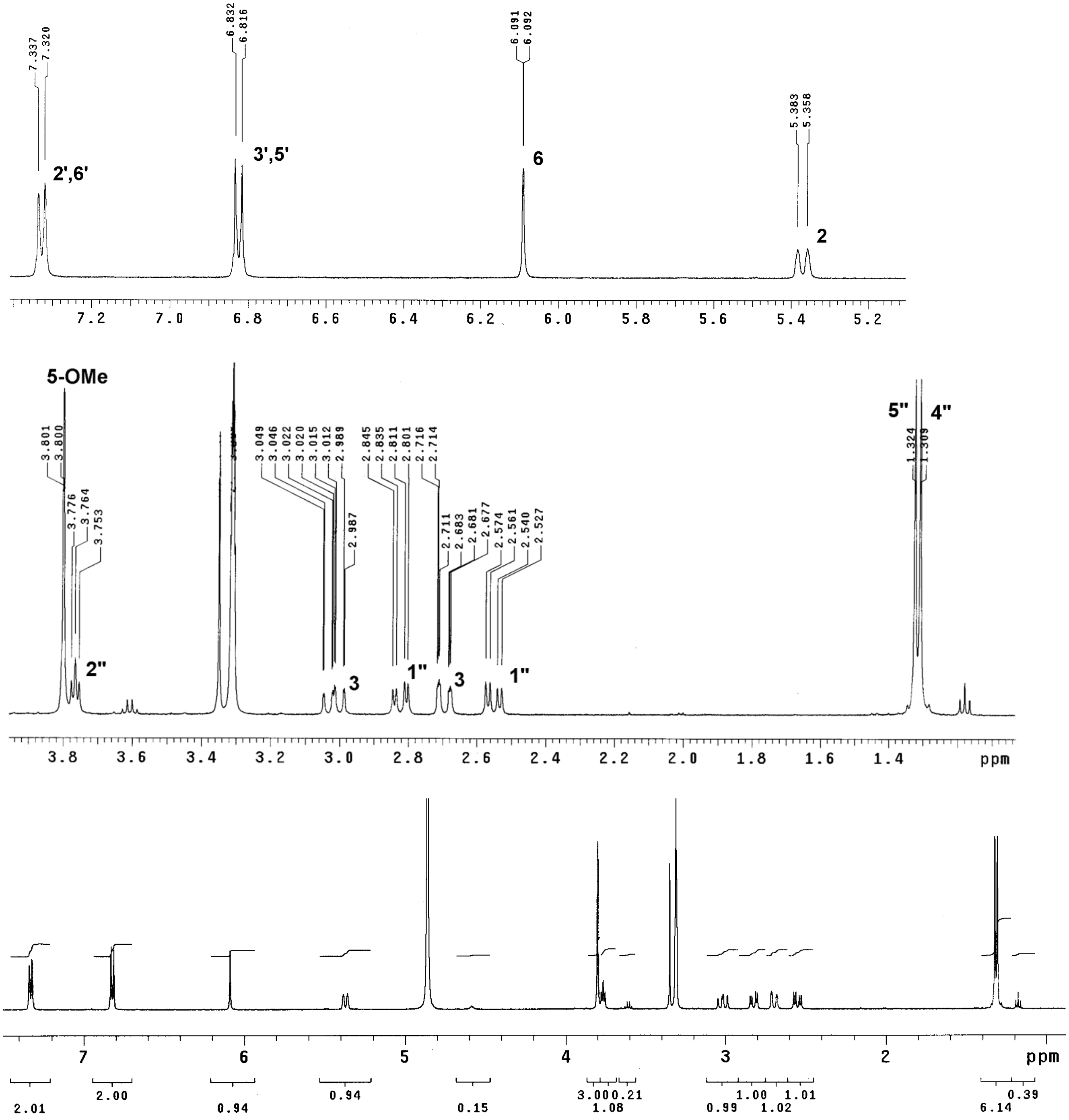

Figure S4. ^{13}C NMR spectrum of **2**

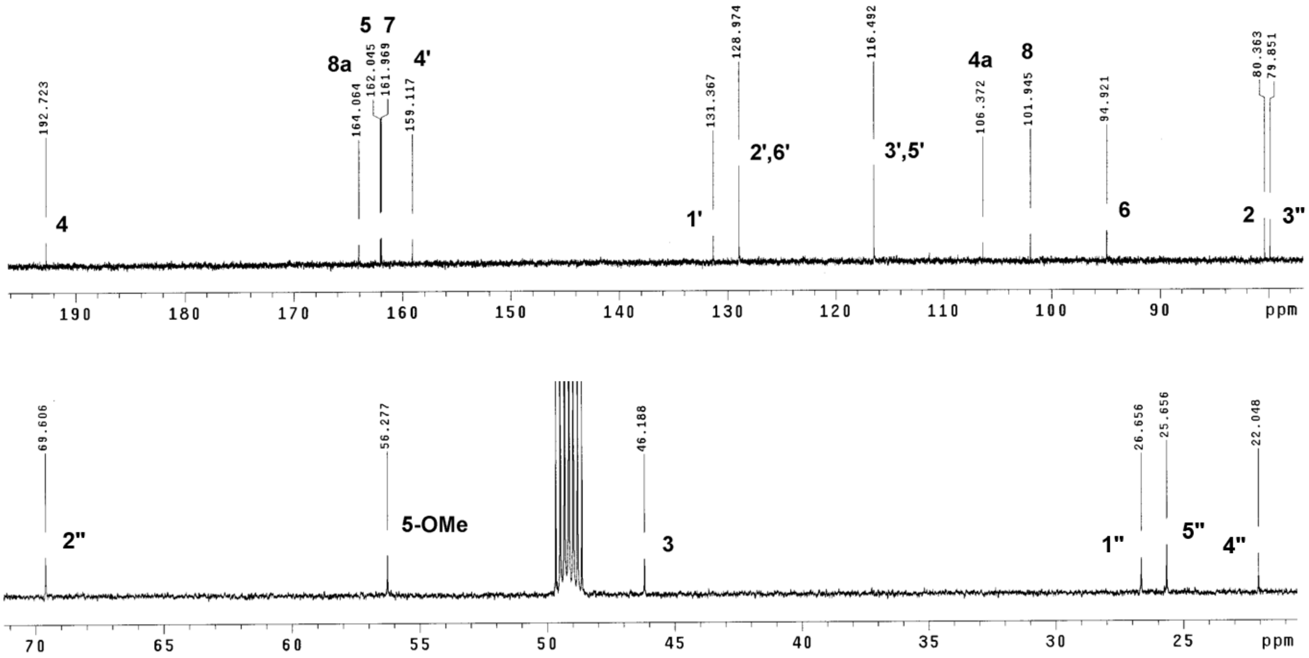

Figure S5. ^{1}H NMR spectrum of the new compound **3**

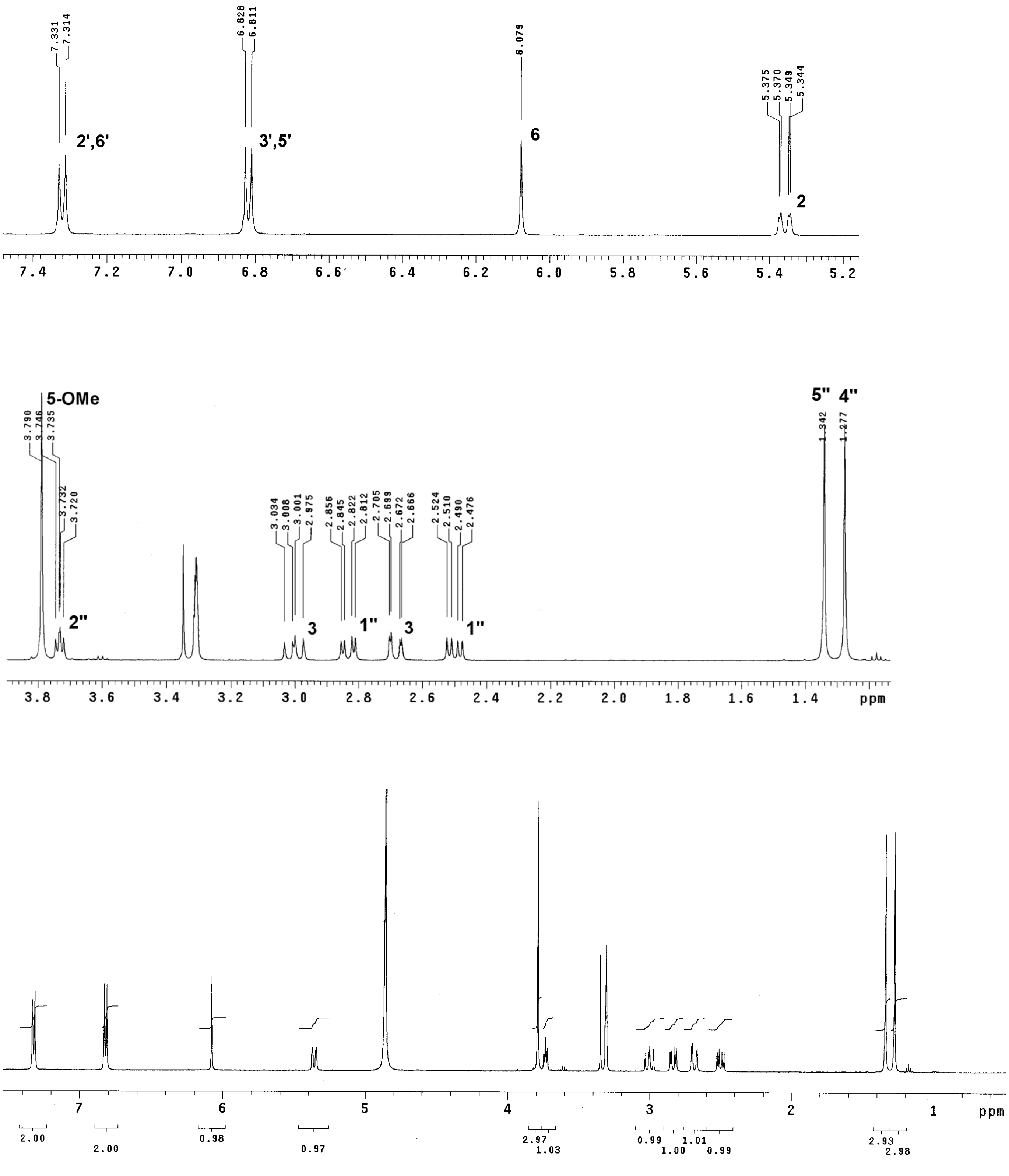

Figure S6. ^{13}C NMR spectrum of the new compound **3**

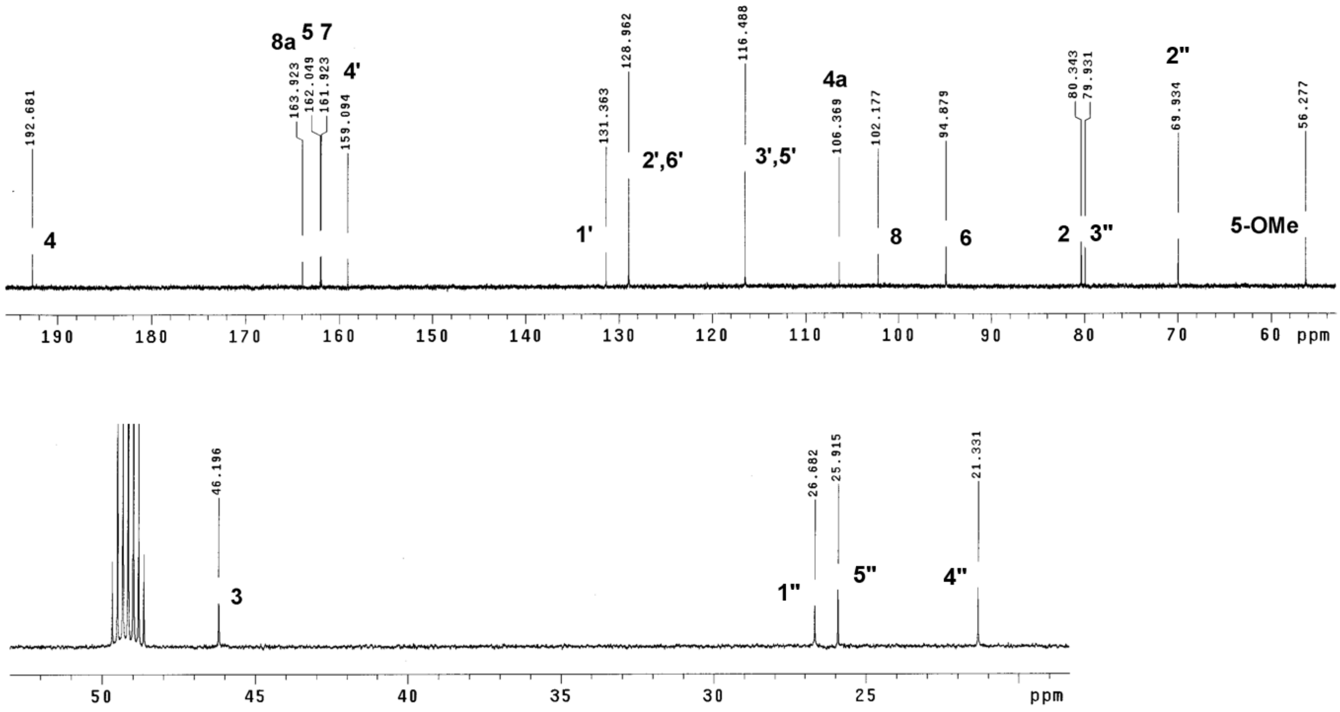

Figure S7. HSQC NMR spectrum of a mixture of **2** and **3**

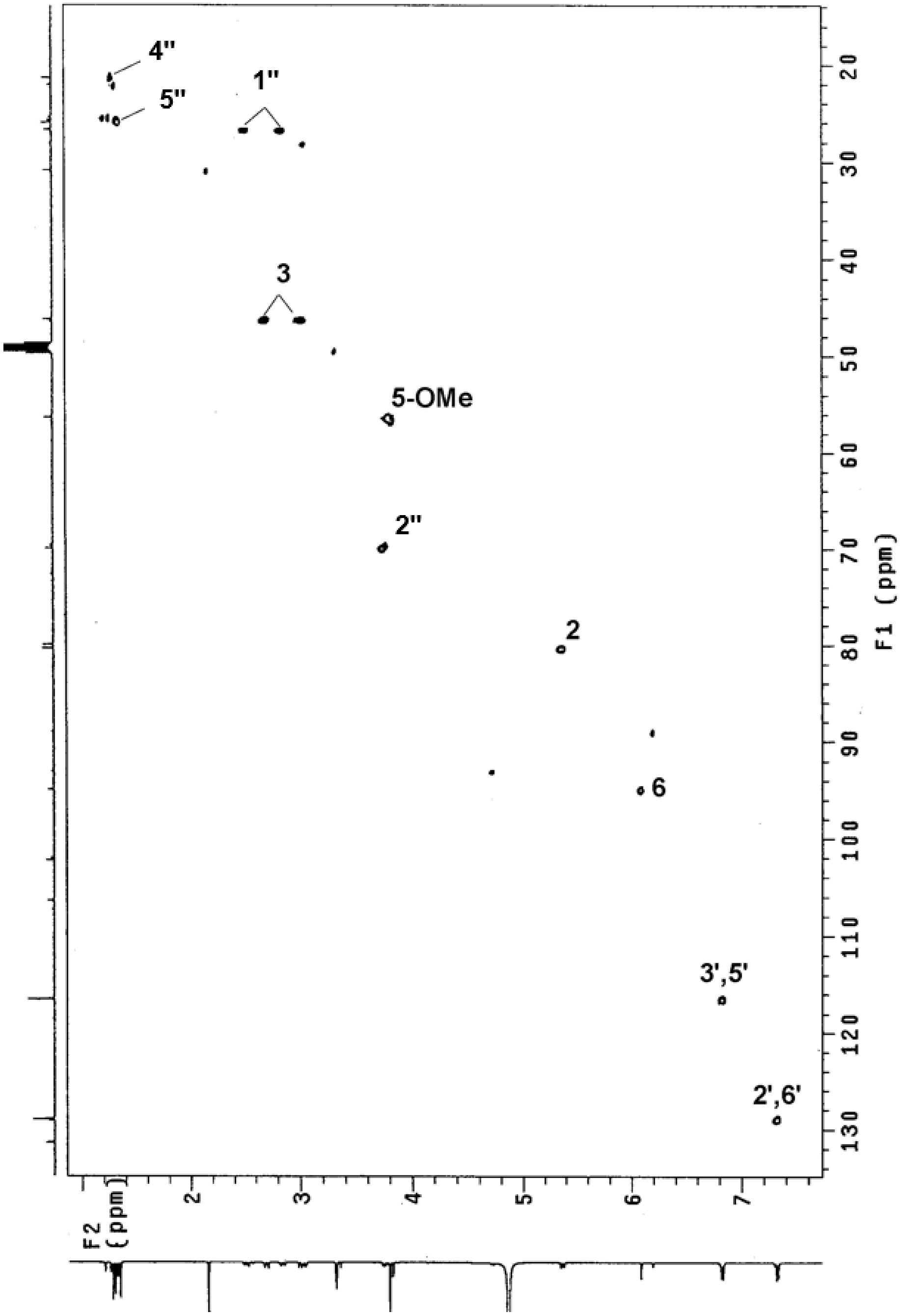

Figure S8. HMBC NMR Spectrum of a mixture of **2** and **3**

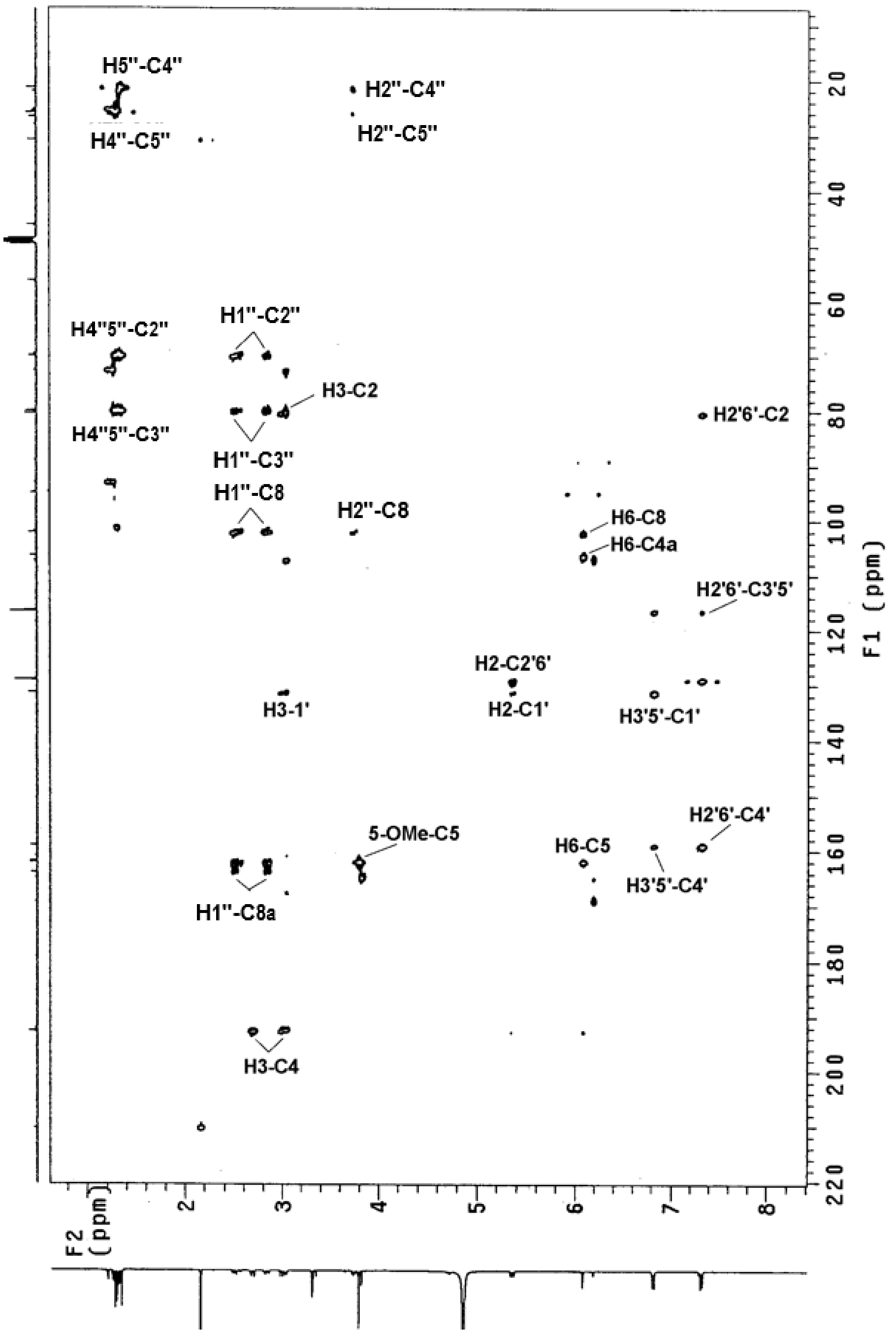

Figure S9. ^{1}H NMR spectrum of the new compound **4**

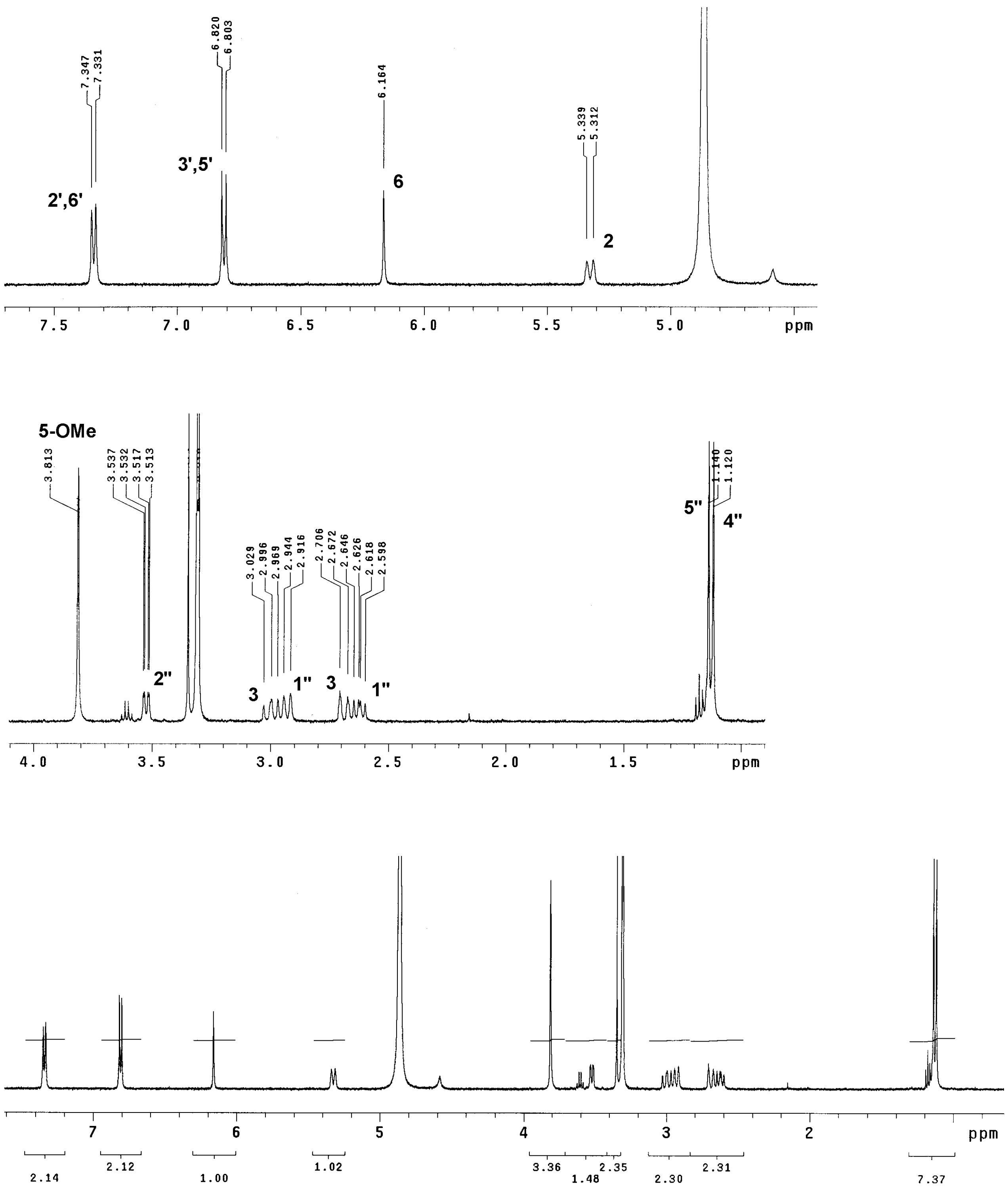

Figure S10. ^{13}C NMR spectrum of the new compound **4**

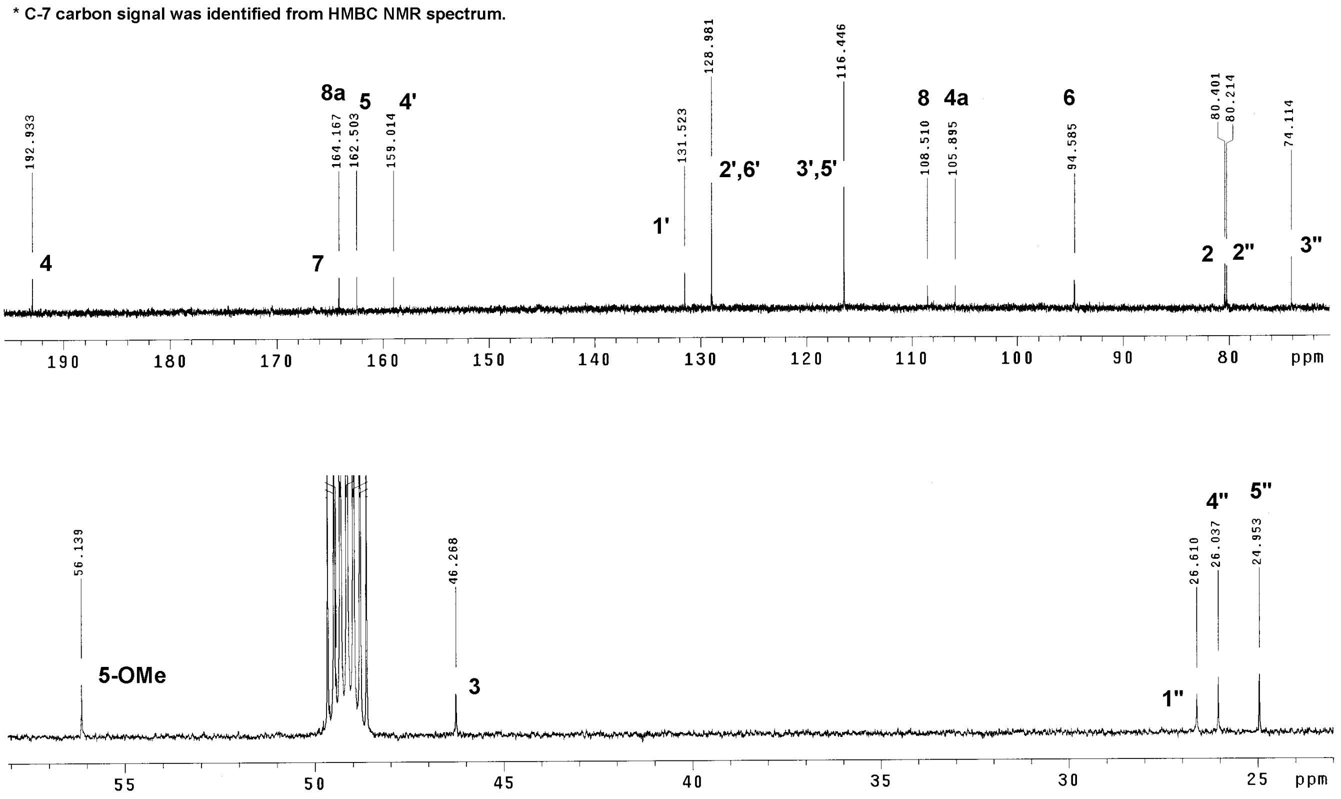

Figure S11. ^1H NMR spectrum of the new compound **5**

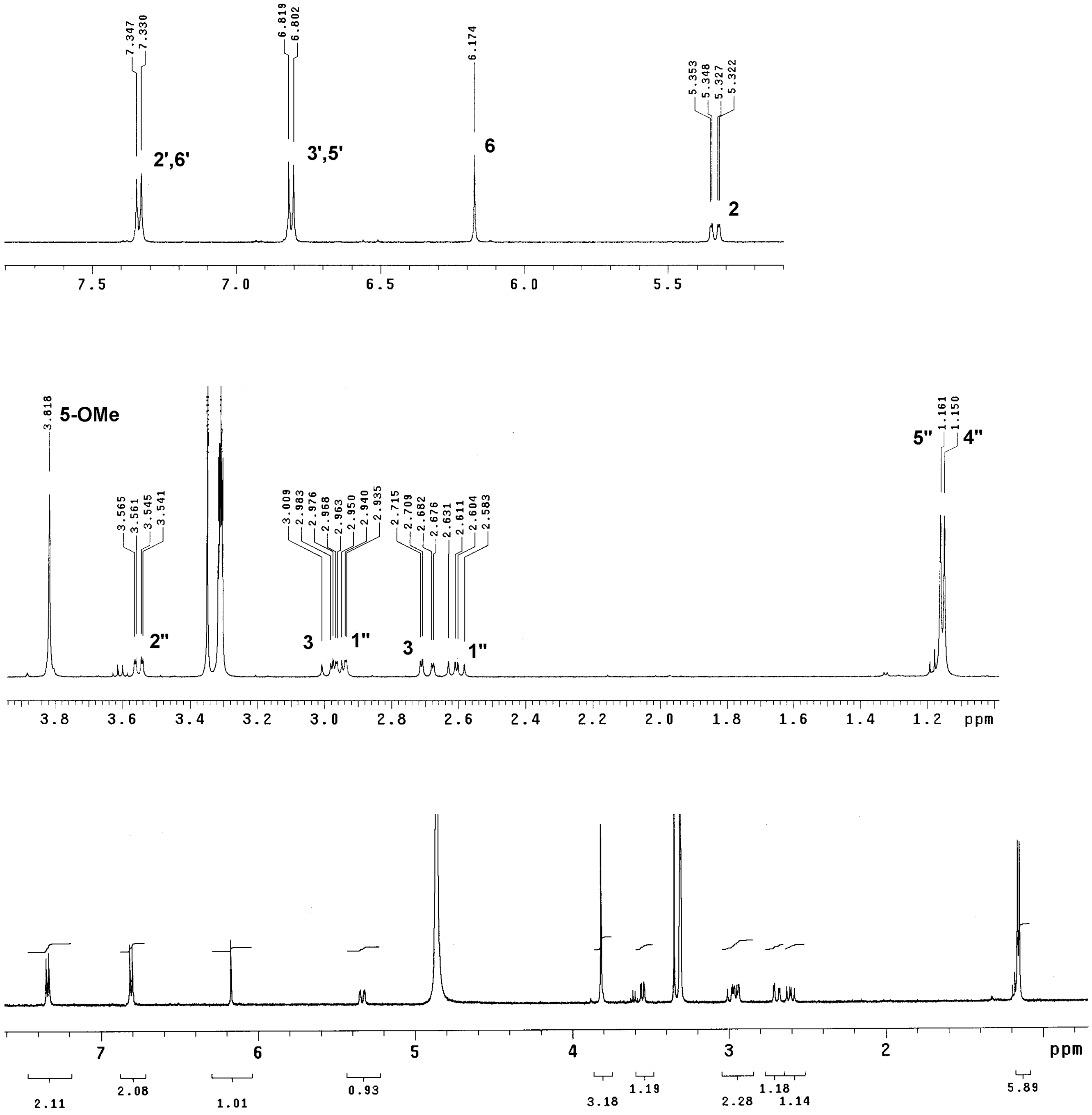

Figure S12. ^{13}C NMR spectrum of the new compound **5**

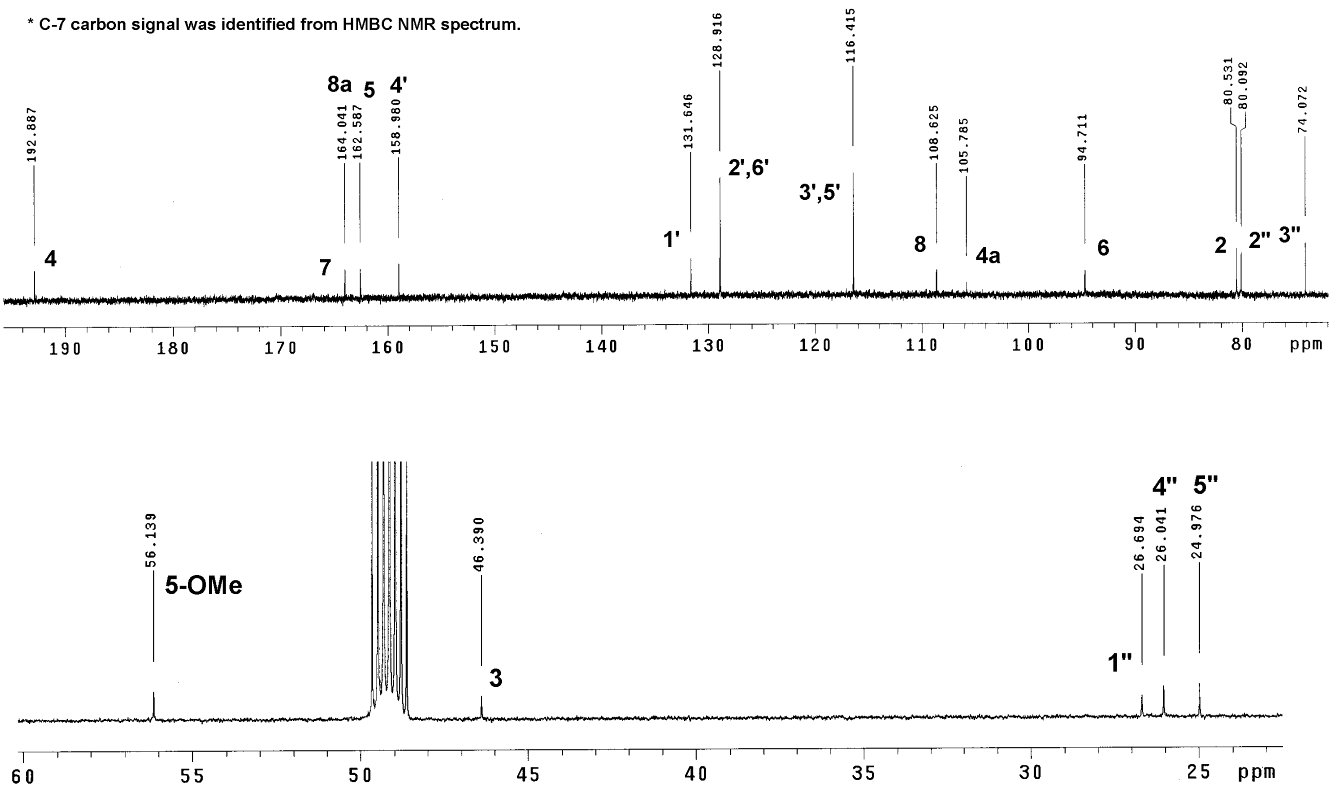

Figure S13. HSQC NMR spectrum of a mixture of **4** and **5**

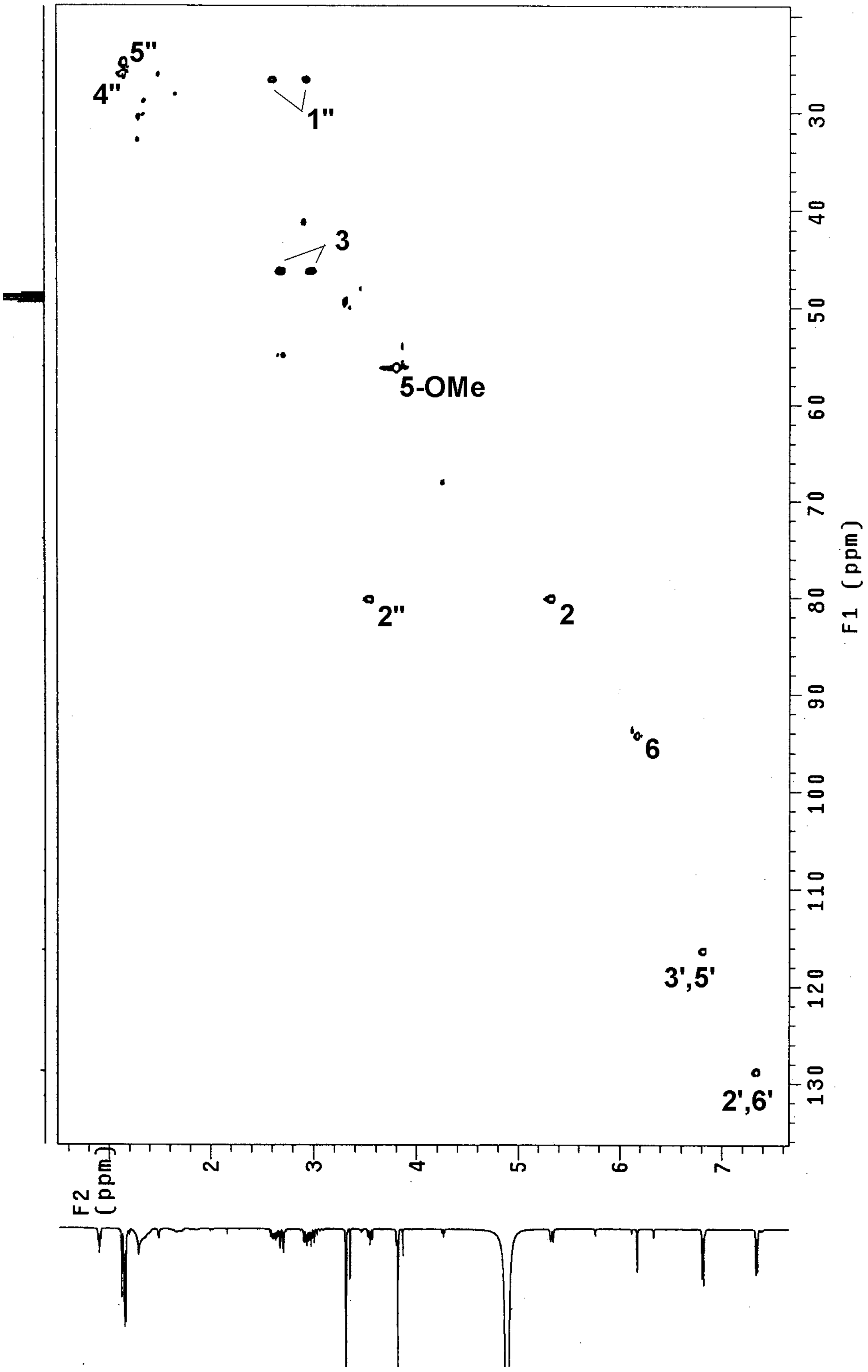

Figure S14. HMBC NMR spectrum of a mixture **4** and **5**

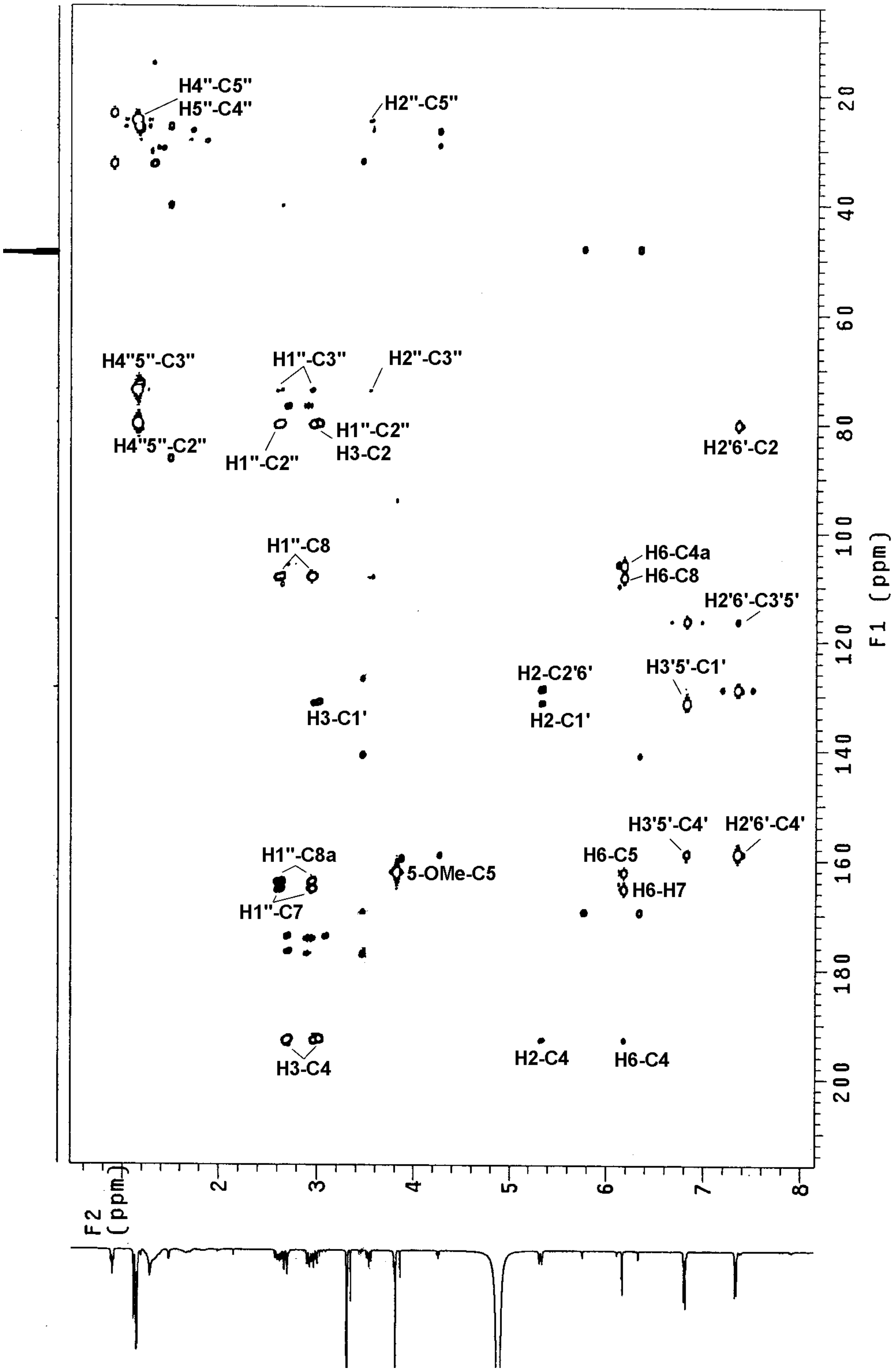

Figure S15. ^{1}H NMR spectrum of **6**

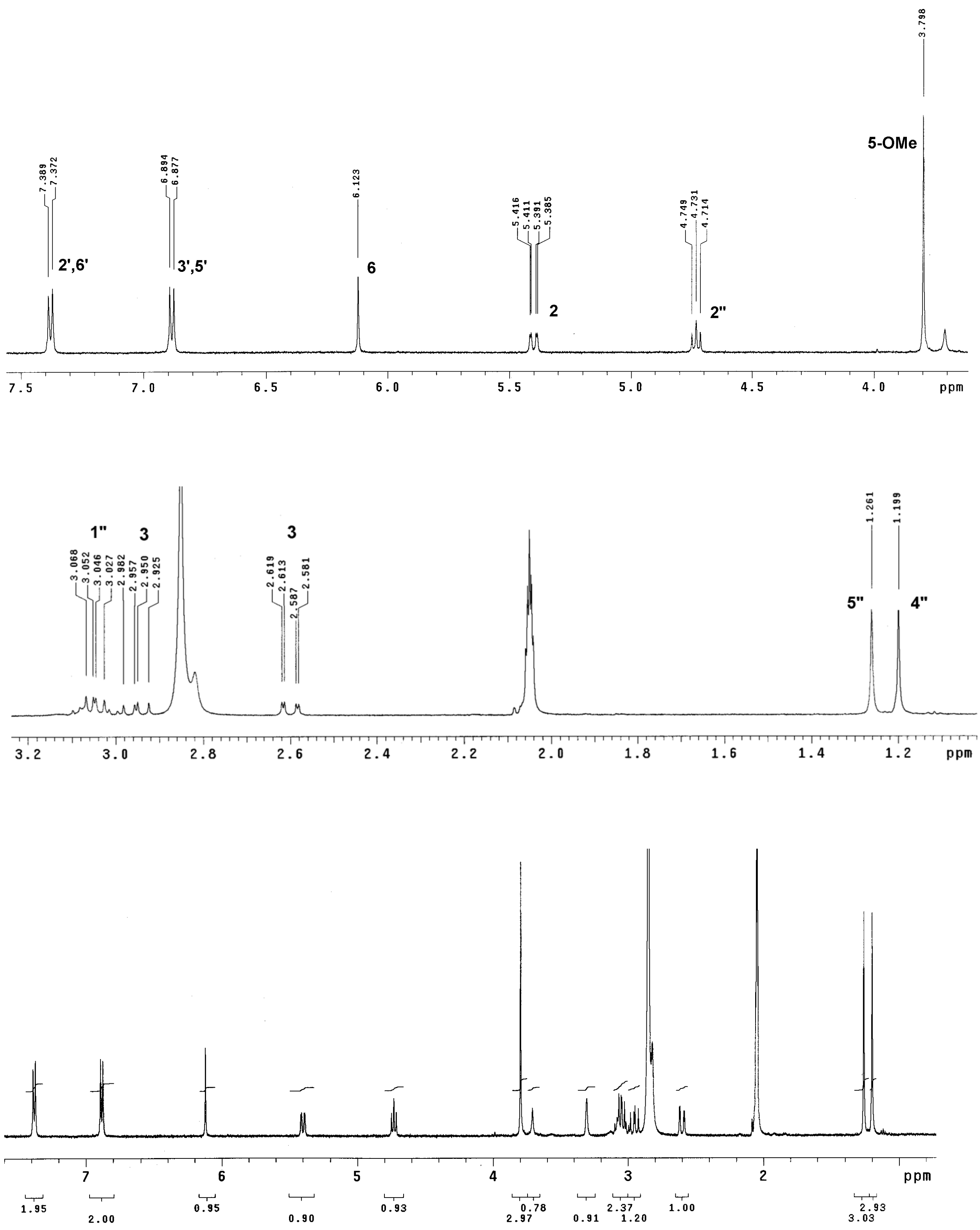

Figure S16. ^{13}C NMR spectrum of **6**

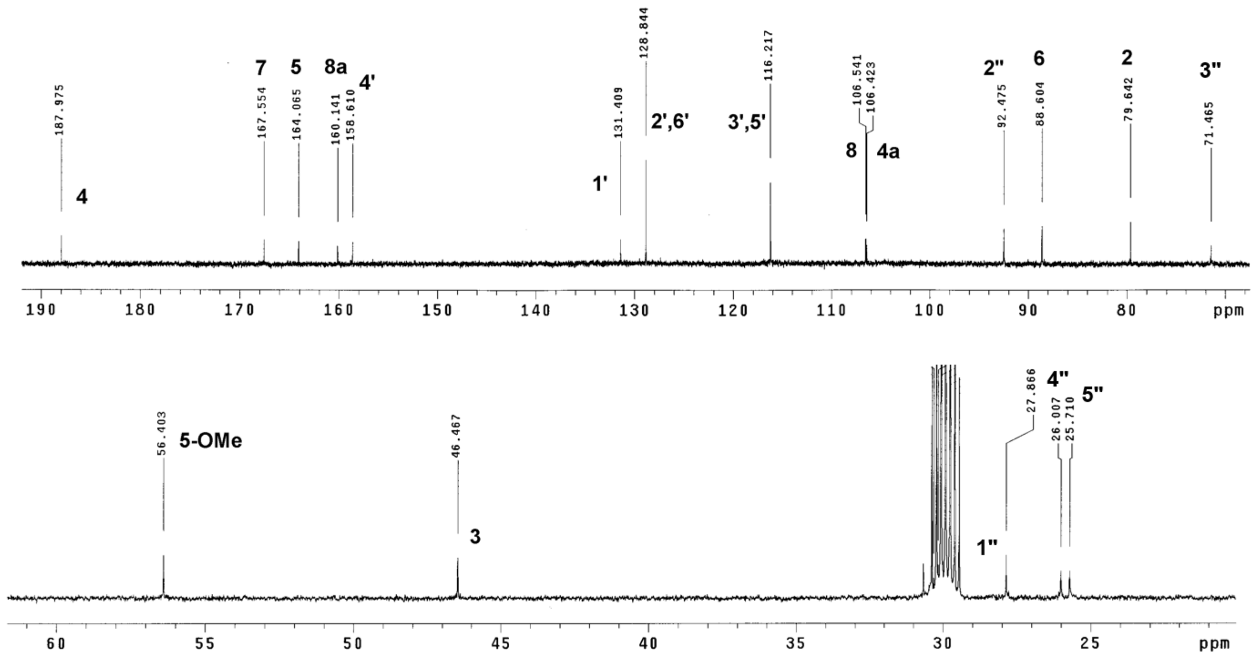

Figure S17. ^{1}H NMR spectrum of **7**

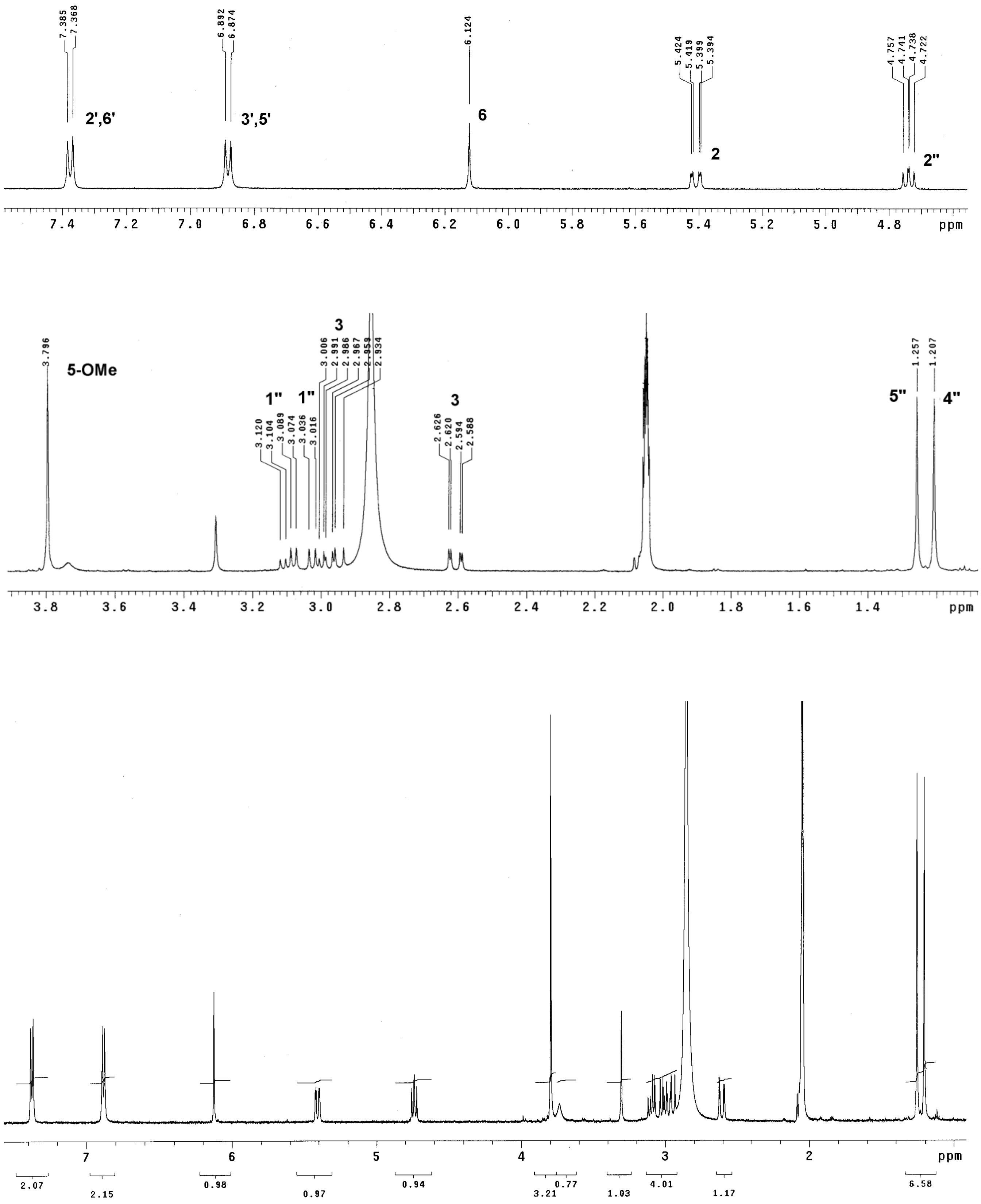

Figure S18. ^{13}C NMR spectrum of **7**

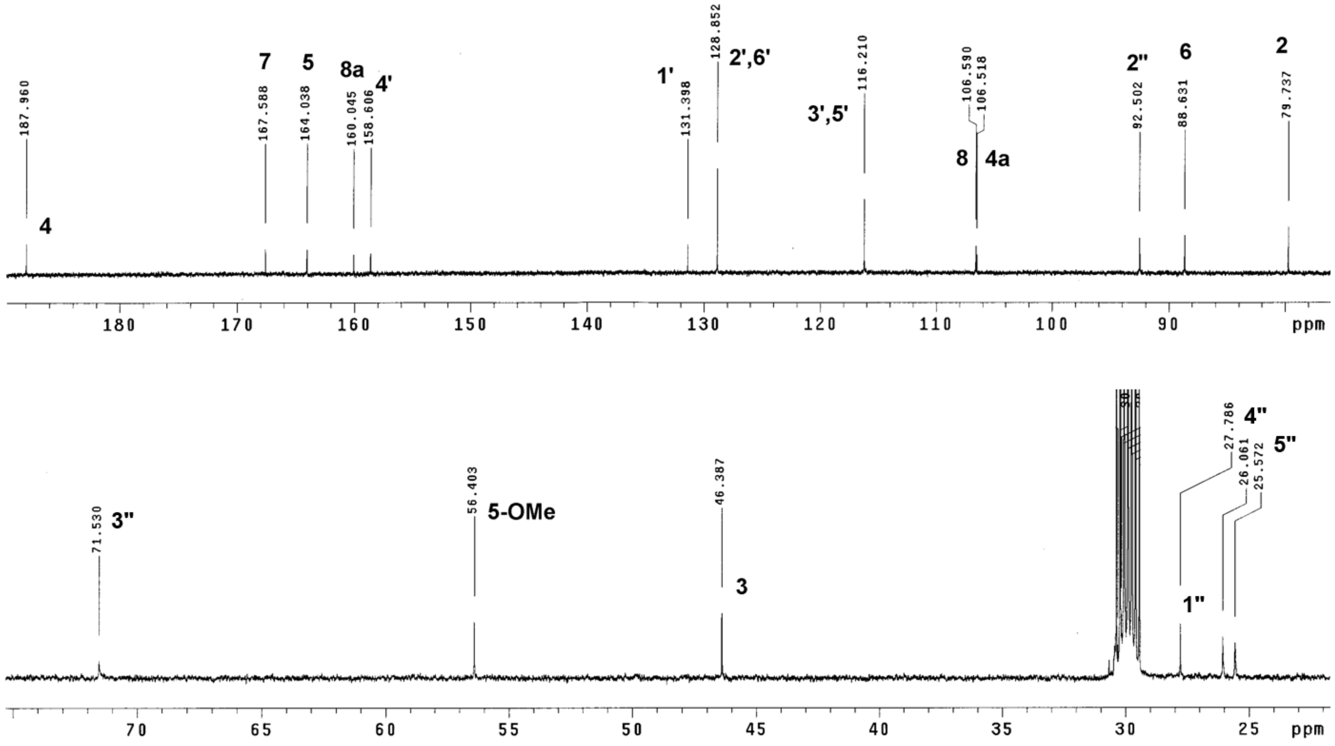

Figure S19. High resolution ESIMS data of metabolites **2**–**7**

a) Metabolite **2**

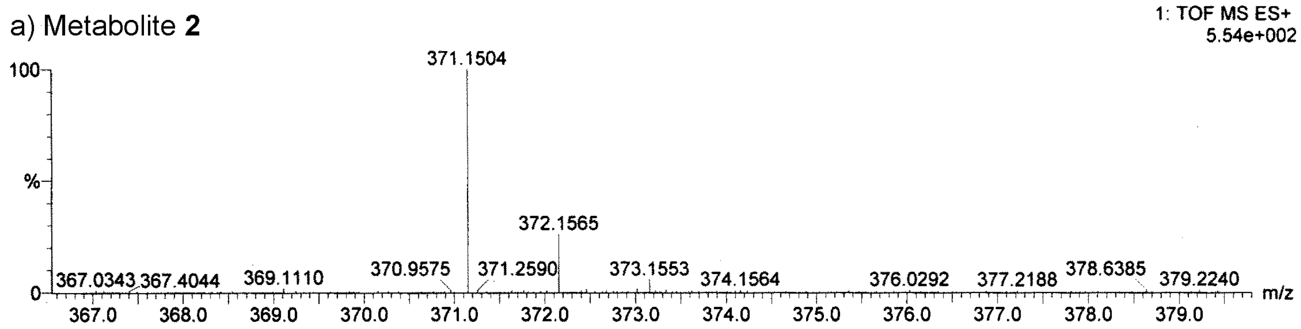

Minimum:				-1.5			
Maximum:		3.0	10.0	50.0			
Mass	Calc. Mass	mDa	PPM	DBE	i-FIT	i-FIT (Norm)	Formula
371.1504	371.1495	0.9	2.4	10.5	92.8	0.0	C21 H23 O6

b) Metabolite **3**

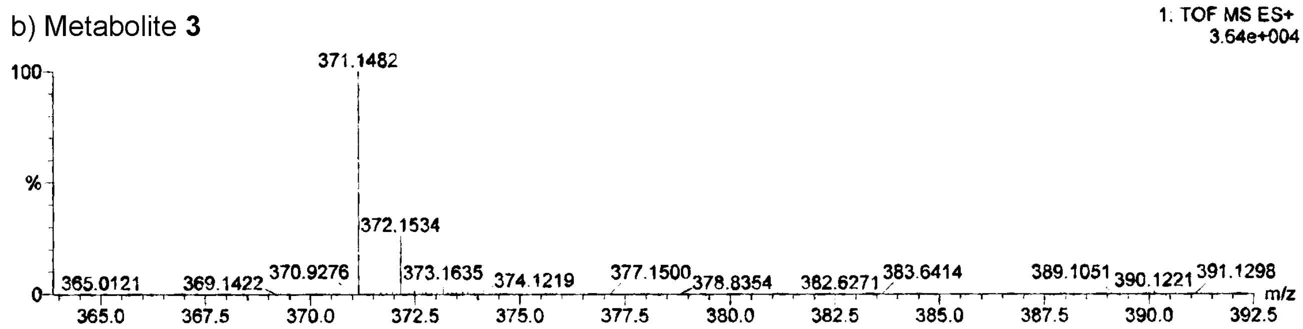

Minimum:				-1.5			
Maximum:		3.0	10.0	50.0			
Mass	Calc. Mass	mDa	PPM	DBE	i-FIT	i-FIT (Norm)	Formula
371.1482	371.1495	-1.3	-3.5	10.5	195.2	0.0	C21 H23 O6

c) Metabolite **4**

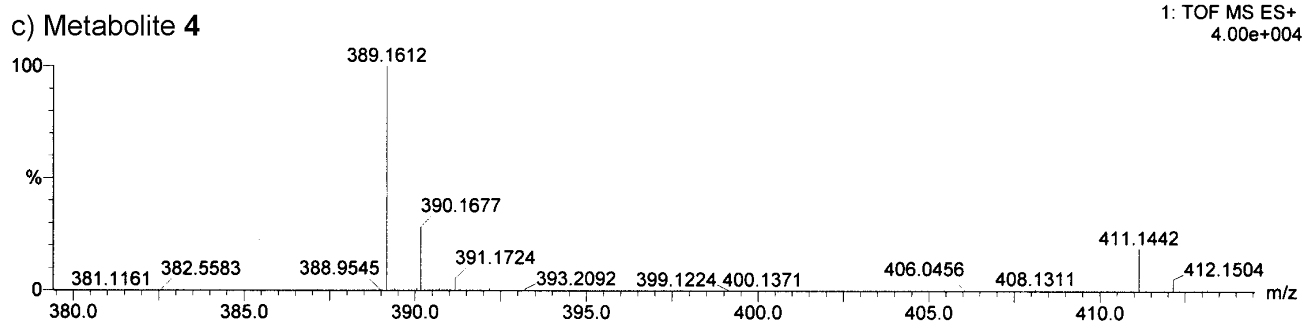

Minimum:				-1.5			
Maximum:		3.0	10.0	50.0			
Mass	Calc. Mass	mDa	PPM	DBE	i-FIT	i-FIT (Norm)	Formula
389.1612	389.1600	1.2	3.1	9.5	341.7	0.0	C21 H25 O7

d) Metabolite **5**

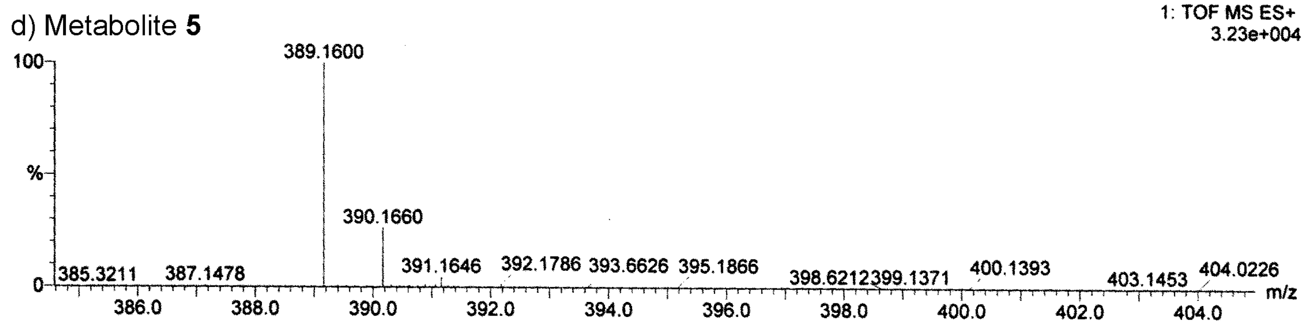

	Mass	Calc. Mass	mDa	PPM	DBE	i-FIT	i-FIT (Norm)	Formula
Minimum:					-1.5			
Maximum:			3.0	10.0	50.0			
	389.1600	389.1600	0.0	0.0	9.5	247.2	0.0	C21 H25 O7

e) Metabolite **6**

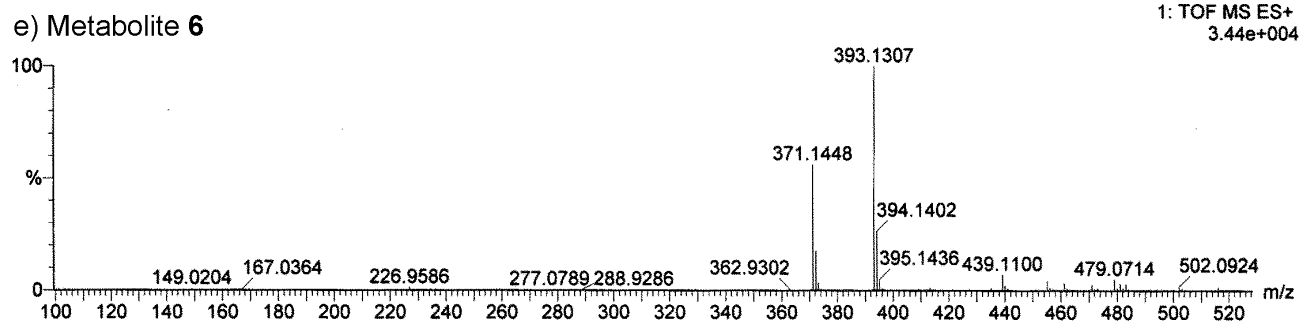

	Mass	Calc. Mass	mDa	PPM	DBE	i-FIT	i-FIT (Norm)	Formula
Minimum:					-1.5			
Maximum:			5.0	10.0	50.0			
	393.1307	393.1314	-0.7	-1.8	10.5	344.2	0.0	C21 H22 O6 Na

f) Metabolite **7**

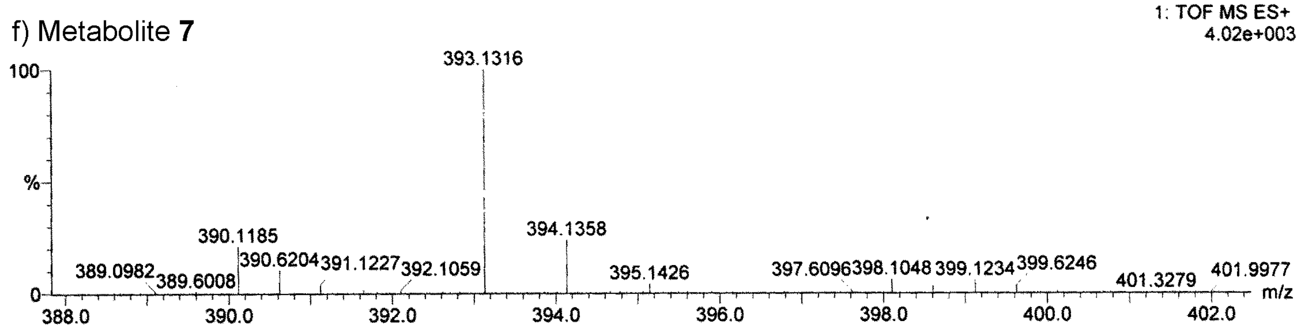

	Mass	Calc. Mass	mDa	PPM	DBE	i-FIT	i-FIT (Norm)	Formula
Minimum:					-1.5			
Maximum:			10.0	10.0	50.0			
	393.1316	393.1314	0.2	0.5	10.5	195.6	0.0	C21 H22 O6 Na

Figure S20. Circular dichroism (CD) profiles of metabolites **2–7**

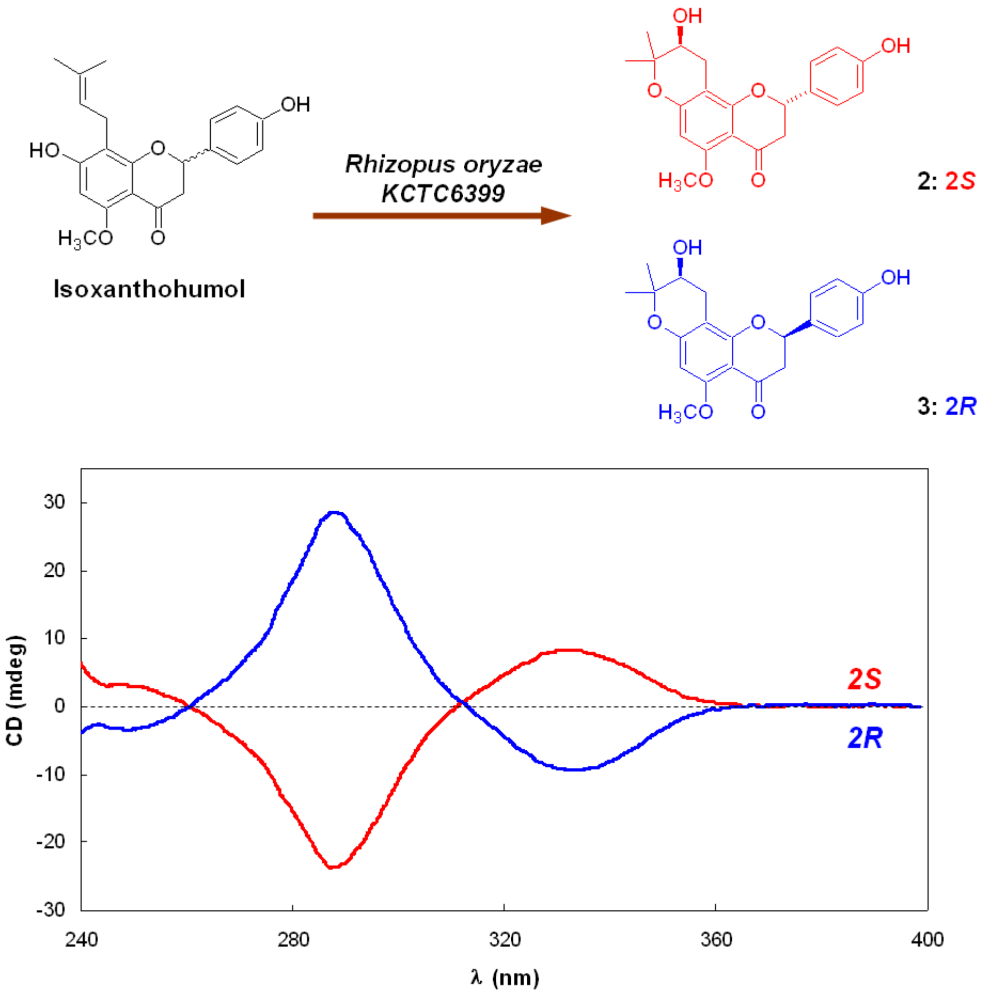

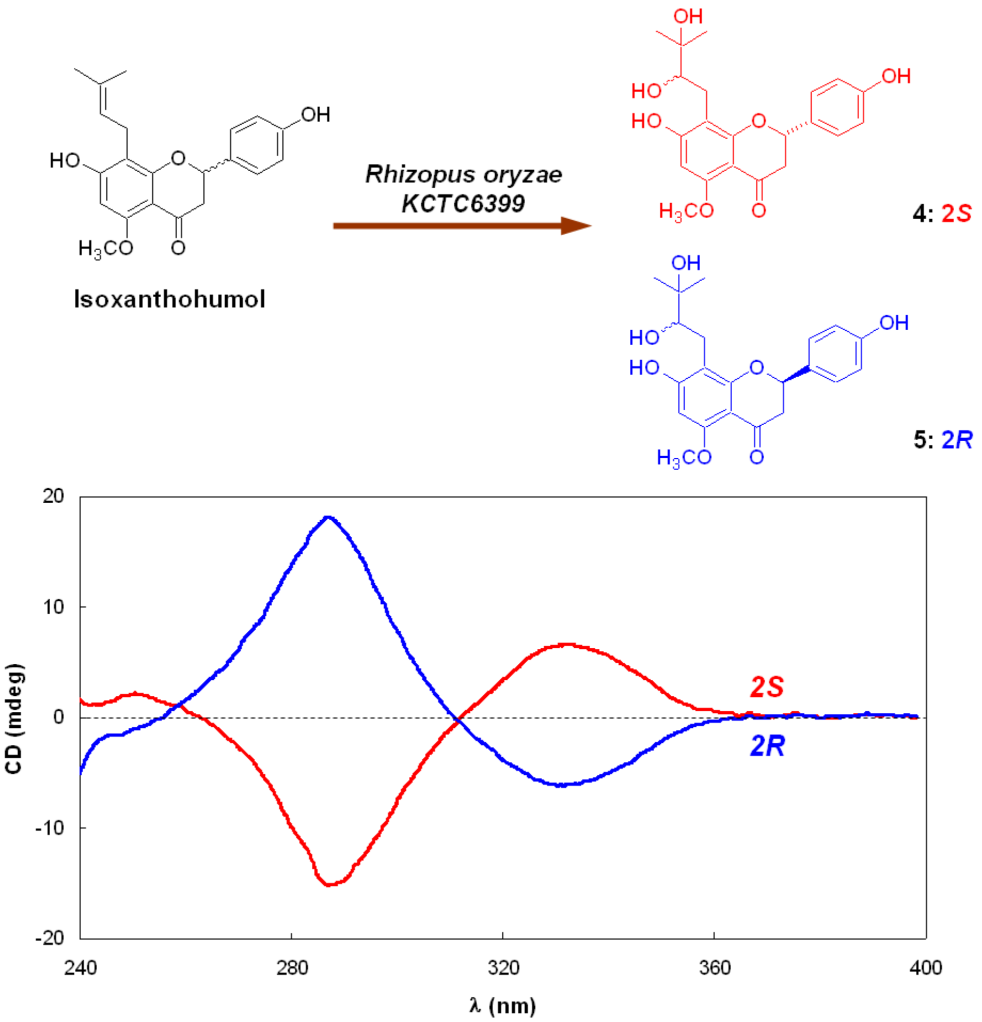

OH
HO
H3CO
O
Isoxanthohumol
Rhizopus oryzae
KCTC6399
4: 2S
5: 2R
20
10
0
-10
-20
CD (mdeg)
240
280
320
360
400
λ (nm)
2S
2R

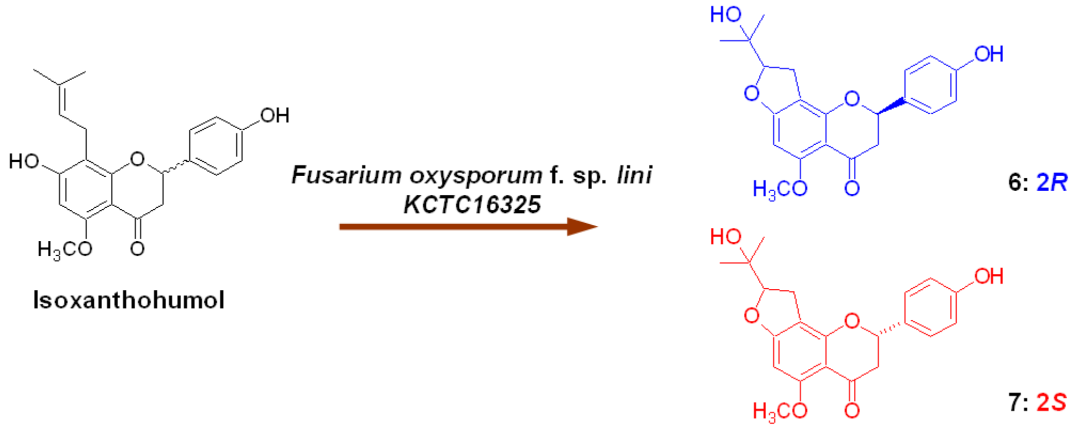
HO
OH
O
H_3CO
O
Isoxanthohumol
Fusarium oxysporum f. sp. *lini*
KCTC16325
6: 2*R*
7: 2*S*

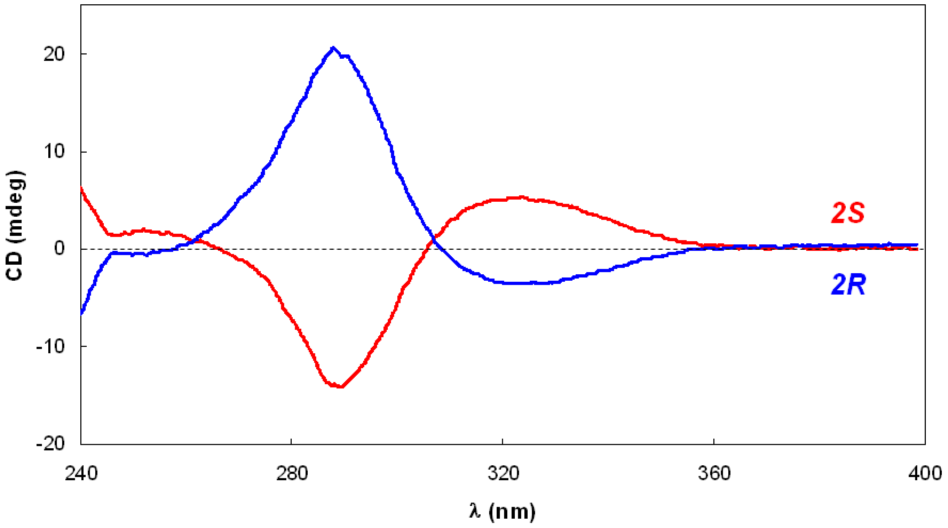
CD (mdeg)
λ (nm)
20
10
0
-10
-20
240
280
320
360
400
2*S*
2*R*
